# Systematic Discovery of Archaeal Transcription Factor Functions in Regulatory Networks through Quantitative Phenotyping Analysis

**DOI:** 10.1128/mSystems.00032-17

**Published:** 2017-09-19

**Authors:** Cynthia L. Darnell, Peter D. Tonner, Jordan G. Gulli, Scott C. Schmidler, Amy K. Schmid

**Affiliations:** a Department of Biology, Duke University, Durham, North Carolina, USA; b Program in Computational Biology and Bioinformatics, Duke University, Durham, North Carolina, USA; c Department of Statistical Science, Duke University, Durham, North Carolina, USA; d Department of Computer Science, Duke University, Durham, North Carolina, USA; University of North Carolina at Chapel Hill

**Keywords:** *Archaea*, functional ANOVA, phenomics, transcription factors

## Abstract

Gene regulatory networks (GRNs) are critical for dynamic transcriptional responses to environmental stress. However, the mechanisms by which GRN regulation adjusts physiology to enable stress survival remain unclear. Here we investigate the functions of transcription factors (TFs) within the global GRN of the stress-tolerant archaeal microorganism *Halobacterium salinarum*. We measured growth phenotypes of a panel of TF deletion mutants in high temporal resolution under heat shock, oxidative stress, and low-salinity conditions. To quantitate the noncanonical functional forms of the growth trajectories observed for these mutants, we developed a novel modeling framework based on Gaussian process regression and functional analysis of variance (FANOVA). We employ unique statistical tests to determine the significance of differential growth relative to the growth of the control strain. This analysis recapitulated known TF functions, revealed novel functions, and identified surprising secondary functions for characterized TFs. Strikingly, we observed that the majority of the TFs studied were required for growth under multiple stress conditions, pinpointing regulatory connections between the conditions tested. Correlations between quantitative phenotype trajectories of mutants are predictive of TF-TF connections within the GRN. These phenotypes are strongly concordant with predictions from statistical GRN models inferred from gene expression data alone. With genome-wide and targeted data sets, we provide detailed functional validation of novel TFs required for extreme oxidative stress and heat shock survival. Together, results presented in this study suggest that many TFs function under multiple conditions, thereby revealing high interconnectivity within the GRN and identifying the specific TFs required for communication between networks responding to disparate stressors.

**IMPORTANCE** To ensure survival in the face of stress, microorganisms employ inducible damage repair pathways regulated by extensive and complex gene networks. Many archaea, microorganisms of the third domain of life, persist under extremes of temperature, salinity, and pH and under other conditions. In order to understand the cause-effect relationships between the dynamic function of the stress network and ultimate physiological consequences, this study characterized the physiological role of nearly one-third of all regulatory proteins known as transcription factors (TFs) in an archaeal organism. Using a unique quantitative phenotyping approach, we discovered functions for many novel TFs and revealed important secondary functions for known TFs. Surprisingly, many TFs are required for resisting multiple stressors, suggesting cross-regulation of stress responses. Through extensive validation experiments, we map the physiological roles of these novel TFs in stress response back to their position in the regulatory network wiring. This study advances understanding of the mechanisms underlying how microorganisms resist extreme stress. Given the generality of the methods employed, we expect that this study will enable future studies on how regulatory networks adjust cellular physiology in a diversity of organisms.

## Abstract (precis)

To ensure survival in the face of stress, microorganisms employ inducible damage repair pathways regulated by extensive and complex gene networks. Many archaea, microorganisms of the third domain of life, persist under extremes of temperature, salinity, and pH and under other conditions. In order to understand the cause-effect relationships between the dynamic function of the stress network and ultimate physiological consequences, this study characterized the physiological role of nearly one-third of all regulatory proteins known as transcription factors (TFs) in an archaeal organism. Using a unique quantitative phenotyping approach, we discovered functions for many novel TFs and revealed important secondary functions for known TFs. Surprisingly, many TFs are required for resisting multiple stressors, suggesting cross-regulation of stress responses. Through extensive validation experiments, we map the physiological roles of these novel TFs in stress response back to their position in the regulatory network wiring. This study advances understanding of the mechanisms underlying how microorganisms resist extreme stress. Given the generality of the methods employed, we expect that this study will enable future studies on how regulatory networks adjust cellular physiology in a diversity of organisms.

## INTRODUCTION

Free-living cells experience frequent stress from the extracellular environment. Transcription factors (TFs) and their regulons of target genes comprise a gene regulatory network (GRN), which functions to alter gene expression dynamically in response to stressful and changing environments. Many environmental conditions are chemically and/or physically inextricable (e.g., oxygen levels and salinity) (1), and different types of stresses can cause similar types of cellular damage (2, 3). For example, both exposure to excess metals and radiation can result in redox imbalance (4). Disparate stressors also elicit similar gene expression programs (5). These observations have led to the hypothesis that GRNs responding to each stressor are highly interconnected (6–8). However, the specific mechanisms that underlie these connected responses remain unclear.

Microorganisms known as extremophiles thrive in environments at the limits of life, representing model systems well suited for understanding how GRNs enable physiological adjustment to strong environmental forces. One group of extremophiles, the hypersaline-adapted archaea, colonize salt lakes where salt concentrations can reach saturation. Fluctuations in temperature and oxygen level, intense radiation, and desiccation/rehydration cycles pose a constant challenge to macromolecular and cellular integrity (9). To respond, archaea use a hybrid system of bacterial-like and eukaryotic-like proteins to regulate transcription. The basal transcriptional machinery resembles that of eukaryotes, including RNA polymerase (RNAP), TATA-binding proteins (TBPs), and transcription factor IIB (TFIIB) homologs (10–12). In contrast, the regulatory proteins are homologous to those found in bacteria, such as TFs containing a helix-turn-helix (HTH) DNA binding domain (13). Many archaeal TFs directly sense environmental changes, binding ligands or changing redox status to alter TF conformation and TF-DNA binding (14).

In the genetically tractable hypersaline-adapted species *Halobacterium salinarum*, GRN inference (7, 15) and subsequent validation experiments suggested that the GRN is required for dynamic adjustment of gene expression in response to extreme and interconnected stress regimes (16–19). Specifically, the integration of transcriptome data in response to environmental and genetic perturbations (1, 7, 15, 17, 19–25), gene functions (26, 27), and *cis*-regulatory motif predictions in the context of statistical inference algorithms (28, 29) resulted in a genome-wide Environment and Gene Regulatory Influence Network (EGRIN) model that predicted regulatory connections for more than 70 TFs and their target genes (7, 15). More recently, similar GRN models have been constructed for other species of archaea (30) and bacteria (31). These models are highly predictive of gene expression in response to stress and enable generation of novel hypotheses regarding the roles of TFs in stress response.

However, for organisms across the domains of life, it remains a central challenge to decipher whether and how genetic and environmental perturbation to the GRN directly impacts cellular phenotype and survival in ecologically relevant contexts (3). Systematic “phenomics” approaches hold promise for understanding the roles of TFs and other regulators in GRNs and how this role impacts cellular physiology (32–34); however, relative to other systems biology methods such as transcriptomics and proteomics, phenomics remains an underrepresented data source.

In response to these challenges, a library of 27 TF deletion mutants was generated in *H. salinarum*. These mutants were assessed for growth under a variety of stresses endemic to the salt flat environment. A novel nonparametric model was developed using a Gaussian process framework to quantify these phenotypes. We used recently developed statistical tests (35) and developed new tests to identify significant differential growth between the trajectories of these TF knockouts relative to that of the control strain. The results revealed that a surprising number of TFs are required for optimal growth under multiple stress conditions, indicating a high level of interconnectivity within the GRN. Clustering analysis of phenotype trajectories revealed that TFs with related phenotypes function together, regulating each other and common sets of genes in stress-specific subnetworks. Through further analysis of TF roles in gene regulation, stress-related functions were validated for novel TFs. We detected strong concordance of newly discovered TF functions with statistical predictions of TF gene regulatory relationships from GRN models inferred from gene expression data alone.

## RESULTS AND DISCUSSION

### Identification of transcription factor candidates.

**FIG 1:**
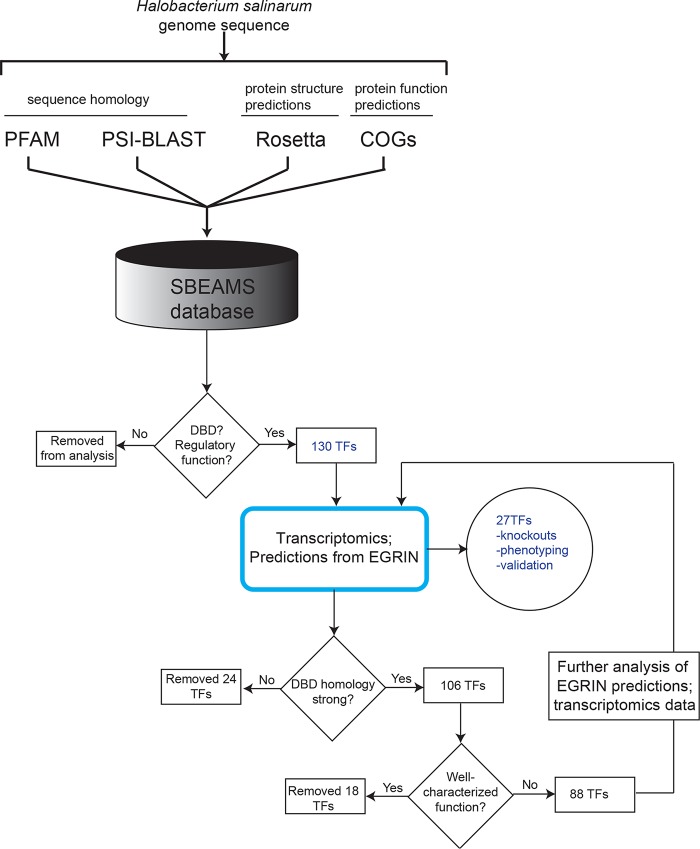
TF candidate selection pipeline. Genes encoding proteins with a putative DNA binding domain were annotated using sequence databases PFAM (37) and PSI-BLAST (38), structural predictions (26), and protein functions from COG (39). These annotations were stored in the Systems Biology Experiment and Analysis Management System (SBEAMS) database (36), resulting in 130 putative TFs. Transcriptome analysis across 1,495 experimental conditions (43) and GRN network inference models (7, 15) were then used to generate predictions regarding TF functions. Details of GRN predictions and criteria for selection of the final collection of 27 TFs are given in Table S1 in the supplemental material. DBD, DNA binding domain.

To prioritize candidate TFs for phenotypic characterization, genes encoding TFs were first detected in the *Halobacterium salinarum* genome (27) using the Systems Biology Experimental Management System database (SBEAMS) (36), where annotations were cross-referenced with other databases (PFAM, COG [clusters of orthologous groups of proteins], and PSI-BLAST [37–39]) (Fig. 1). Previous *ab initio* protein structure prediction results (26) and subsequent matches to the Protein Data Bank (PDB) were used to identify possible DNA binding domains in proteins of unknown function. This pipeline resulted in a list of 130 putative TFs, which were included as priors in inference of the *H. salinarum* EGRIN model (7, 15). To identify candidates of interest for further analysis here, TFs were more stringently defined as those with strong homology (E value ≤ 1 × 10^−7^) to a sequence-specific DNA binding domain or structural fold experimentally characterized in other bacterial or archaeal species (13, 26) (Fig. 1). This definition excluded 24 DNA-binding proteins with peripheral roles in transcription (e.g., helicases). Another 18 genes encoding previously published, well-characterized TFs, were also excluded (21, 40–42). Of the 88 remaining TFs, a final subset of 27 were selected for further study based on transcriptional changes during fluctuations in a wide array of environmental conditions (1, 7, 20, 23–25, 43) and functional predictions from EGRIN gene regulatory network models (7, 15) (Fig. 1; see Table S1 in the supplemental material). The TFs in the resultant collection are members of a variety of functional families and contain diverse structural domains (Table S1). These include DNA binding domains known from other archaea and bacteria (winged helix-turn-helix, ribbon-helix-helix) and domains unique to halophilic archaea (e.g., HalX). Some TFs contain ligand binding domains homologous to those of known function in bacteria, such as DtxR family iron-dependent repressors. Other TFs possess domains of novel function specific to halophiles, such as the RosR C-terminal domain (16) (Table S1). Together, the results of these bioinformatic analyses suggest that the panel of selected TFs is representative of the global transcription regulatory landscape of *H. salinarum*.

10.1128/mSystems.00032-17.7 TABLE S1  Details on bioinformatic analysis for selecting 27 TFs of interest in this study. Download TABLE S1, XLSX file, 0.04 MB. Copyright © 2017 Darnell et al. 2017 Darnell et al. This content is distributed under the terms of the Creative Commons Attribution 4.0 International license.

### Quantification of significant differential growth of TF knockout strains under stress. (i) Experimental design and data.

To determine the physiological function for each TF and systematically compare these functions across TFs, knockout mutant strains for each of the 27 selected TFs were grown under five conditions: standard growth, low salinity, paraquat (PQ), hydrogen peroxide, and heat shock (see Materials and Methods). The growth of these knockout mutant strains was compared to that of the isogenic parent control strain, Δ*ura3* strain, a uracil auxotroph used to generate knockout mutants (44) (see Materials and Methods). Growth conditions were chosen based on their relevance to the hypersaline habitat of *H. salinarum*. Knockout mutants for 10 of these 27 TFs were constructed in previous studies, where the role of each TF was assessed under a single stress condition (Table 1). These strains were included for the following reasons: (i) as a control to validate our methods and (ii) to test possible secondary functions for these previously studied regulators. Strains with deletion of the genes encoding the remaining 17 TFs of interest were constructed in the current study using established genetic methods for *H. salinarum* (see Materials and Methods) (44) (Table S2). The genotypes of all 27 mutants, regardless of prior publication, were also verified here (see Materials and Methods; Fig. S1 and Table S2). Growth of each knockout and parent control strain was measured under each of the five conditions every 30 min for 48 h, resulting in 210,180 data points (raw data given in Table S3).

10.1128/mSystems.00032-17.8 TABLE S2  Lists of primers, plasmids, and strains used in this study. Download TABLE S2, XLSX file, 0.02 MB. Copyright © 2017 Darnell et al. 2017 Darnell et al. This content is distributed under the terms of the Creative Commons Attribution 4.0 International license.

10.1128/mSystems.00032-17.1 FIG S1  PCR confirmation of TF mutants. See Table S2 for the primers and PCR conditions. All PCR products are diluted to correct for DNA concentration and the resulting band brightness. +, PCR product from the Δ*ura3* strain; −, product from the mutant strain indicated at the top. Marker sizes are indicated to the left. Download FIG S1, PDF file, 0.4 MB. Copyright © 2017 Darnell et al. 2017 Darnell et al. This content is distributed under the terms of the Creative Commons Attribution 4.0 International license.

10.1128/mSystems.00032-17.9 TABLE S3  Raw growth data for 27 TF knockout mutants under the five treatment conditions. These data were used as input to the FANOVA growth model. Download TABLE S3, XLSX file, 3.5 MB. Copyright © 2017 Darnell et al. 2017 Darnell et al. This content is distributed under the terms of the Creative Commons Attribution 4.0 International license.

**TABLE 1:** Strains used in this study with known phenotypes and functions, and types of evidence previously generated for each strain

Condition	Strain(s)	References	Knockout growth	Gene expression	TF-DNA binding	Metabolomics
Oxidative stress	Δ*rosR*, Δ*VNG0194H*, Δ*hrg*, Δ*sirR*, Δ*asnC*	16, 35, 50, 54	*✓*	*✓*	*✓*	
Nutrient acquisition	Δ*trmB*	17, 18, 48, 49	*✓*	*✓*	*✓*	*✓*
Manganese and iron homeostasis	Δ*idr*1, Δ*idr*2, Δ*sirR*	19, 22	*✓*	*✓*	*✓*	
Copper overload	Δ*copR*	22, 54	*✓*	*✓*	*✓*	

### (ii) Development of a FANOVA model and test for differential growth of knockout mutants under stress.

**FIG 2:**
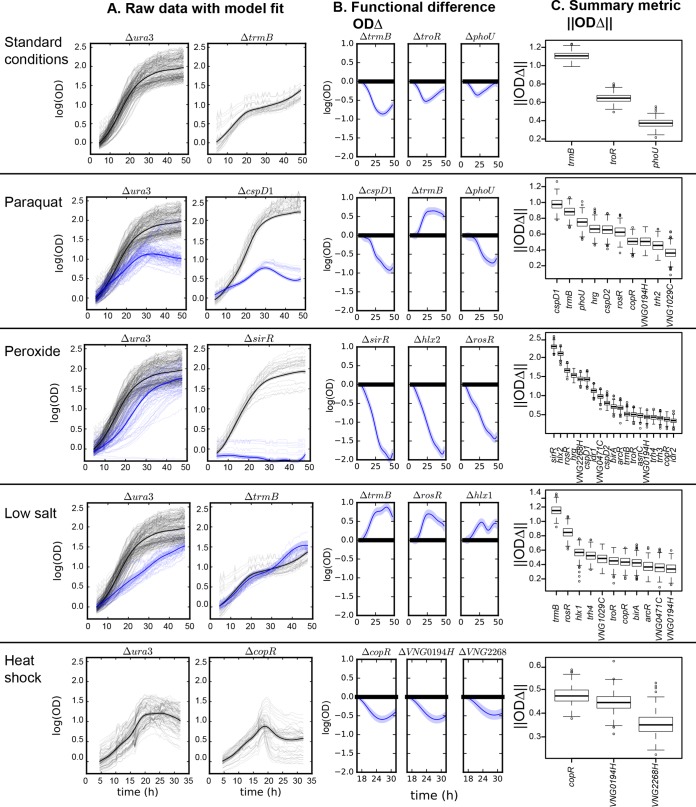
FANOVA modeling and statistical ranking of TF knockout mutant growth phenotypes across five environmental conditions, standard growth conditions, paraquat stress, peroxide, low salt, and heat shock. (A) Mutants with the largest difference in growth compared to the Δ*ura3* control strain under each condition. Raw data from growth trajectories for individual cultures under standard conditions (thin gray lines) were fit and compared with growth under stress conditions (thin blue lines) using FANOVA (see Materials and Methods). Solid lines indicate the mean of the fit to all replicate trajectories, and shaded regions are the 95% confidence interval of the fit. (B) Functional difference (OD_Δ_) of the TF knockout strain relative to the isogenic Δ*ura3* parent strain. The mutants with the top three scoring phenotypes according to $||{\text{OD}}_{\Delta }||$ are shown. (C) Summary statistical metric ranking ($||{\text{OD}}_{\Delta }||$) for those mutants with strongly different growth trajectories compared to that of the Δ*ura3* strain ($||{\text{OD}}_{\Delta }||$≥ 0.337 across all conditions).

Typically, microbial population growth is modeled using parametric models, such as Gompertz regression (45). The effects of stress and genetics on growth are then quantified by testing for statistically significant differences in the estimated parameters for different conditions (46). However, we previously showed that the impact of genetics and stress perturbations on growth are more accurately captured with a nonparametric Gaussian process (GP) model (35). In contrast to parametric models, GPs have the advantage of learning these relations directly from the data and do not require explicit equations describing the effects of stress and genetics on growth, which are typically unknown in the case of phenotypic discovery. In this study, we again use GPs to model microbial population growth, now in the form of functional analysis of variance (FANOVA) (47). In the FANOVA setting, microbial population growth is divided into a linear combination of different experimental variables: condition, genetic background, and the interaction between the two (see Materials and Methods) (Fig. 2A). Additionally, FANOVA allows for the explicit comparison of different effects in terms of significance, even if the shapes of those effects are quite different. For these reasons, FANOVA is particularly well adapted to modeling and comparing the effects of many different stress and genetic perturbations. This extends our previous GP model (35) by including all data from multiple conditions and genetic backgrounds into a single model, which allows for direct comparison of different effects in a common framework.

Two metrics were used to assess the significance of differential growth based on our FANOVA model of growth data. The first metric, change in the optical density (OD_Δ_) (35), is a function representing the difference in growth levels between the mutant strain [*f*_*m*_(*x*)] and parent strain [*f*_*p*_(*x*)] over the duration of the growth curve under a specific condition (Fig. 2B and Fig. S2) as follows:

(1) $${\text{OD}}_{\Delta }(t)=f_{m}(t)-f_{p}(t)$$

10.1128/mSystems.00032-17.2 FIG S2  Phenotype trajectories for all 27 TF knockout mutants under all five growth conditions tested. Each plot depicts the mean OD_Δ_ (dark blue line) over the growth time course for each TF knockout with 95% confidence interval (shaded light blue region). The relevant genotypes of the strains are indicated above each graph. Download FIG S2, JPG file, 0.9 MB. Copyright © 2017 Darnell et al. 2017 Darnell et al. This content is distributed under the terms of the Creative Commons Attribution 4.0 International license.

Where the OD_Δ_ curve differs significantly from zero (based on a 95% credible interval), the growth phenotype is considered significantly different from the parent strain for that section of the growth curve. The second metric, $||{\text{OD}}_{\Delta }||$, represents the overall magnitude of the functional difference between parent and mutant strains under a specific condition and is calculated based on OD_Δ_ (Fig. 2C and Fig. S3):

(2) $$||{\text{OD}}_{\Delta }||=\sqrt{\overset{t_{n}}{\underset{t=t_{0}}{\sum }}[{\text{OD}}_{\Delta }(t){]}^{2}}$$

**FIG 3:**
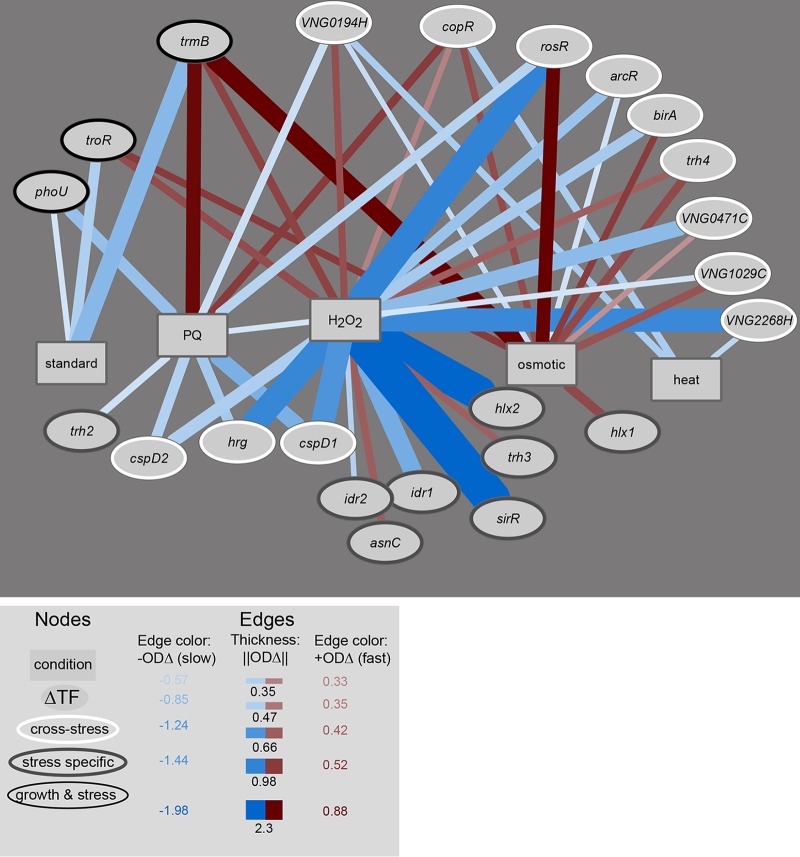
Phenotype network analysis reveals three major classes of TF mutants and extensive cross-regulation of stress responses by TFs. Node and edge attributes are given in the key at the bottom of the figure. OD_Δ_ numbers in the edge color legend refer to the maximum (fast growth) or minimum (slow growth) value of the mean of the posterior prediction from the FANOVA model across the entire growth time course. $||{\text{OD}}_{\Delta }||$numbers for edge thickness refer to the median value of the distribution from $||{\text{OD}}_{\Delta }||$ boxplots (see Fig. 2C, Fig. S3, and Materials and Methods).

10.1128/mSystems.00032-17.3FIG S3 Distribution of $||{\text{OD}}_{\Delta }||$ values for all mutants across conditions. The right and left boundaries of the blue boxes in each graph represent the first and third quartiles, respectively. Red lines represent median values, which were used to determine edge widths in the phenotype network in Fig. 3. Download FIG S3, PDF file, 1 MB.Copyright © 2017 Darnell et al.2017Darnell et al.This content is distributed under the terms of the Creative Commons Attribution 4.0 International license.

Higher values of $||{\text{OD}}_{\Delta }||$ indicate an overall larger deviation from parent strain behavior, regardless of positive or negative growth phenotype, and were used as a rank ordering of phenotype severity to prioritize TF mutants for further analysis.

### (iii) FANOVA modeling of microbial population growth enables the discovery of new TF knockout phenotypes.

On the basis of $||{\text{OD}}_{\Delta }||$ ranking, the Δ*trmB* strain exhibited the strongest growth impairment under standard conditions (Fig. 2A). This result was expected based on previous characterization of TrmB as a global regulator of nutrient metabolism (17, 18, 35, 48, 49). Also as expected from prior work, the Δ*rosR* mutant with deletion of a key oxidative stress response TF was among the top-ranking quartile of mutants under oxidative stress (16, 35, 50). Consistent with prior Gaussian process model results, the Δ*sirR* strain exhibited a significant growth impairment under peroxide stress (35); indeed, here the Δ*sirR* strain exhibited the top-ranking peroxide phenotype of all mutants tested (Fig. 2C). The consistency of these results with those of prior studies demonstrates the validity of the FANOVA model for recapitulating known phenotypes.

Surprisingly, however, several novel mutant phenotypes were observed. For instance, the Δ*cspD1* mutant showed the strongest growth impairment relative to the parent control strain under PQ stress, and the Δ*copR* mutant exhibited the strongest growth impairment under heat shock (Fig. 2A and B). In order to compare the number of significant mutant phenotypes under each condition, a universal cutoff of $||{\text{OD}}_{\Delta }||$ of 0.337 was chosen (see Materials and Methods). This cutoff allowed rank ordering of the stress conditions themselves in terms of the strength of perturbation to physiology, as measured by the number of significant mutant phenotypes. Peroxide treatment had the strongest effect, with 19 mutants exhibiting significant differences in growth compared to the Δ*ura3* strain (12 mutants grew more slowly than the Δ*ura3* strain, and 7 mutants grew faster than the Δ*ura3* strain [Fig. S2 and S3]). The next strongest effect was low salt (11 mutants, 9 faster and 2 slower than the Δ*ura3* strain), followed by PQ (10 mutants, 2 faster and 8 slower than the Δ*ura3* strain), heat shock and standard conditions (3 mutants in each condition, all with significantly impaired growth phenotypes [Fig. 2C]). We conclude that FANOVA analysis recapitulates known roles of TFs but also suggests novel contributions of TFs to cell physiology.

### Phenotype network analysis reveals extensive cross-regulation of stress responses by TFs.

Many mutants exhibited significant differential growth phenotypes under multiple conditions tested (Fig. 2). At the rank order $||{\text{OD}}_{\Delta }||$ cutoff (Fig. 2C), 23 of the 27 mutants studied exhibited significant differential growth under at least one condition (Fig. 3). Network analysis of these phenotypes across conditions enabled the classification of the mutants into three categories. (i) Significant differential growth was detected for 12 mutants relative to the Δ*ura3* strain under two or more stress conditions. These TFs were considered to have “cross-stress” functions (Fig. 3). (ii) Significant differential growth was detected for three mutants (Δ*trmB*, Δ*phoU*, and Δ*troR* mutants) under both standard conditions and one or more stress conditions. These TFs were considered to have “growth & stress” functions. (iii) Eight TFs were required for normal growth under only one of the conditions tested here and therefore considered “stress-specific” TFs. However, three of the stress-specific mutants also exhibited growth defects under other stress conditions tested *ad hoc* in previous studies but not included in the systematic phenomic testing here (e.g., Δ*sirR*, Δ*idr1*, and Δ*idr2* mutants are required for metal homeostasis [19, 22]), suggesting that some of these “stress-specific” TFs might also be required for protection across multiple stressors.

Several mutants with strong growth impairments relative to the parent control strain under one condition exhibited growth improvement under others. For example, although the Δ*trmB* mutant grew poorly under standard conditions, it showed significantly improved growth under PQ and osmotic stress conditions (maximum OD_Δ_ of 0.5 and 1.0, respectively; Fig. 2A and 3). Similarly, the Δ*rosR* mutant exhibited substantially improved growth under low osmolarity (maximum OD_Δ _of 0.8) but strong growth defects under oxidative stress induced by PQ and peroxide. Deletion of the gene encoding a third TF, CspD1, led to increased growth relative to the control strain under standard conditions but impaired growth under oxidative stress (Fig. 2A and 3 and Fig. S2). These observations suggest novel functions for these previously characterized TFs. These opposing phenotypic patterns for individual TFs could result from direct regulation of genes required for growth and/or stress resistance under these conditions. For example, ribosome levels are directly related to growth rate across the tree of life (51, 52), including archaea (25), and *H. salinarum* RosR directly regulates ribosome biosynthesis genes (50). Alternatively, alteration in growth rate *per se* could change cellular stress resistance properties; for example, slow growth in wild-type yeast cells is associated with heat shock resistance (53). Together, the classes of TF mutants identified here suggest that a surprisingly high number of TFs are required for growth homeostasis during exposure to multiple stressors, suggesting extensive network interconnectivity.

### The structure of the gene regulatory network corresponds strongly with TF physiological functions.

**FIG 4:**
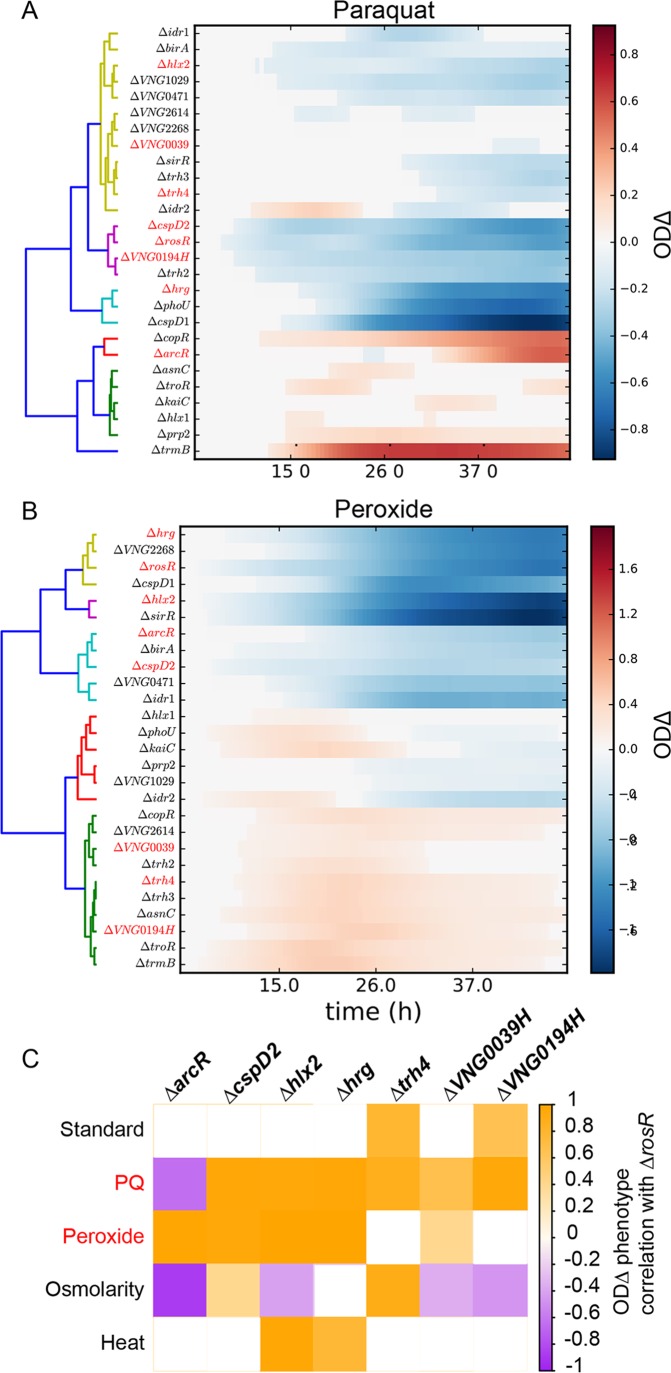
TFs that regulate each other have similar OD_Δ_ phenotype trajectories. (A and B) Heat maps depict hierarchical clustering of OD_Δ_ trajectories for the 27 TF knockout mutants under paraquat (PQ) (A) and peroxide (B) conditions. TFs transcriptionally regulated by RosR are known (50) and indicated by red text. Colors in the dendrogram represent different clusters. The color scale indicates the mean of the posterior OD_Δ_ distribution for each mutant across the growth time course (*x* axis). (C) Each of the seven mutants regulated by RosR are statistically enriched for strongly correlated phenotypes ($\hat{\rho }$≥ 0.4) with the Δ*rosR* mutant under PQ and standard growth conditions relative to other conditions (red text). Colored squares represent significant correlations (*P* ≤ 0.001), and white squares represent nonsignificant correlations. See the color scale to the right of the figure for correlation values.

To gain insight into possible regulatory mechanisms underlying such phenotypes, we determined the relationship between growth trajectories by clustering phenotypes according to the OD_Δ_ metric (Fig. 4) and asked how correlated phenotypes mapped to GRN topology. In particular, the Δ*rosR* mutant was previously shown to regulate 20 other TFs, 7 of which were included in the strain collection evaluated here (Δ*arcR*, Δ*cspD2*, Δ*hlx2*, Δ*hrg*, Δ*trh4*, Δ*VNG0039H*, and Δ*VNG0194H* strains). Hierarchical clustering of OD_Δ_ phenotypes across the five conditions revealed that these mutants clustered closely together under oxidative stress, including PQ (Fig. 4A) and peroxide conditions (Fig. 4B). Under PQ stress, correlation of the OD_Δ_ trajectories for these mutants with that of the Δ*rosR* mutant were significantly enriched for strong positive correlations (ρ^≥0.4) relative to all other pairwise correlations between the 27 mutants (*P* ≤ 6.90 × 10^−3^ by the hypergeometric distribution test [Fig. 4C]). Under peroxide stress, according to$||{\text{OD}}_{\Delta }||$, knockouts in genes encoding TFs regulated by RosR were also significantly enriched for impaired growth relative to all other mutants (*P* ≤ 0.034 by the hypergeometric distribution test). Such significant phenotype correlations were not observed under other conditions tested (Fig. 4C and Fig. S4), suggesting that the interconnection between these TFs is coordinated specifically under oxidative stress (50). Together, these results are consistent with the hypothesis that TFs that regulate each other in GRN subnetworks controlling the cellular response to a particular stress are predictive of TF knockout phenotypes and vice versa.

10.1128/mSystems.00032-17.4 FIG S4  Heat maps of clustering of OD_Δ_ growth trajectories of all 27 mutants under standard (A), low-salt (B), and heat shock (C) conditions. Color scale of OD_Δ_ values is shown at the right side of the clustered growth trajectories. Colors indicate coherent clusters at the tree height cutoff indicated where blue branches end. Download FIG S4, PDF file, 0.7 MB. Copyright © 2017 Darnell et al. 2017 Darnell et al. This content is distributed under the terms of the Creative Commons Attribution 4.0 International license.

Previous predictions of TF functions from computational GRN models specific to oxidative stress for *H. salinarum* (15) were compared with phenotypic results obtained here. Of the 27 knockouts studied, 15 were predicted by network analysis to play a role in gene regulation during oxidative stress (15). Of these 15, 14 TF knockouts showed significantly altered growth relative to the Δ*ura3* control in oxidative stress induced by PQ, peroxide, or both (Fig. 2C, Fig. S3, and Table S1). This suggests that the GRN computationally predicted from gene expression data has strong predictive power for the roles of TFs in cell physiology.

### Validation and characterization of novel TF functions. (i) CopR functions as a regulator of both heat shock and copper overload responses.

**FIG 5:**
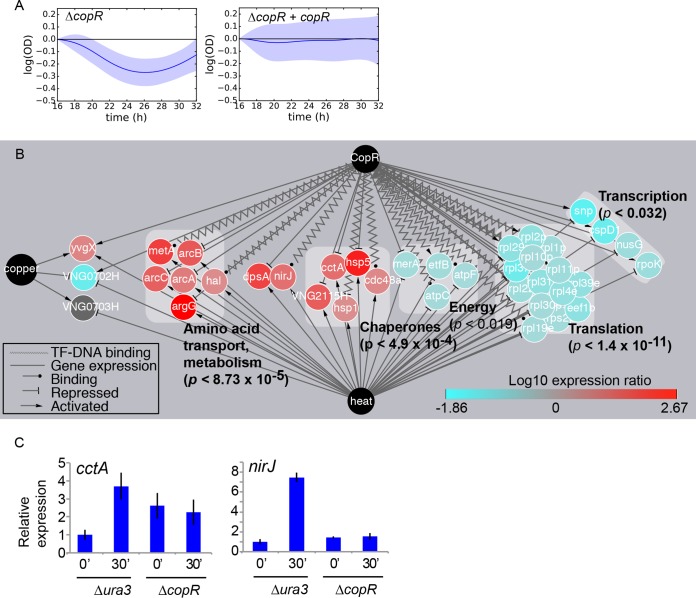
Phenotype validation: a novel heat shock function for the copper-responsive regulator CopR. (A) Slow growth under heat shock conditions in the Δ*copR* mutant (left graph) is complemented by expression of the *copR* gene in *trans* (right graph). (B) Cytoscape gene regulatory network depicting the significant overlap between genes regulated by CopR in response to copper overload (CopR node at the top) and in wild-type cells in response to heat shock (heat node at the bottom) (55) and copper (copper node on the left) (22). Node colors (representing expression levels in wild-type cells under heat shock) and edge line types are indicated in the keys. Gray boxes behind groups of nodes represent arCOG functional categories (61). *P* values of enrichment were calculated by the hypergeometric distribution test. Genes of unknown function are not shown here for clarity but are given in Fig. S5. (C) Quantitative RT-PCR gene expression of *cctA* and *nirJ* genes in the knockout strain compared to the Δ*ura3* control strain. The levels of expression before heat shock (0′) and 30 min after induction of heat shock (30′) are shown. Expression is normalized relative to a control gene whose expression does not change during heat shock (see Materials and Methods). Error bars represent standard errors of the means (SEM) of three biological replicate cultures, each with three technical replicate trials.

To support the stress-specific novel functions for individual TFs discovered here, we performed validation experiments. First we tested the observation of secondary functions for the previously characterized TF, CopR (Fig. 3). We detected significantly impaired growth of the Δ*copR* mutant relative to the Δ*ura3* parent strain during heat shock (Fig. 2A). The heat shock phenotype of the Δ*copR* mutant was the top-ranking mutant under this condition (Fig. 2C). These observations were surprising given that CopR (previously called VNG1179C [22, 54]) was previously characterized as a repressor of P1-type ATPases that export copper during overload (22). To conduct further functional validation of the role for CopR in response to elevated temperature, a wild-type copy of the *copR* gene was expressed in *trans* on a plasmid in the Δ*copR* deletion background, which returned the growth of this strain to levels indistinguishable from that of the Δ*ura3* parent control (Fig. 5A). This complementation result indicates that the Δ*copR* heat shock growth defect was caused by loss of the *copR* gene alone and not by polar or off-target secondary site genetic effects.

10.1128/mSystems.00032-17.5 FIG S5  Cytoscape network graphs depicting the expression of genes whose expression is affected by heat shock, copper, and deletion of *copR*. (A) Colors represent expression of genes in wild-type cells under heat shock. Levels of expression are indicated in the color key. Gray nodes are not differentially expressed. Edges are indicated as in Fig. 4. Gray boxes surrounding groups of nodes represent arCOG functional groups with enrichment significance *P* values provided. Categories and *P* values are consistent across subpanels. (B) The colors of the nodes represent expression of genes in the Δ*copR* strain. (C) The colors of the nodes represent gene expression under copper overload conditions in wild-type cells. Download FIG S5, PDF file, 1 MB. Copyright © 2017 Darnell et al. 2017 Darnell et al. This content is distributed under the terms of the Creative Commons Attribution 4.0 International license.

To detect potential gene targets of CopR regulation under heat shock, existing transcriptomic and TF-DNA binding data sets were reanalyzed. In the wild-type *H. salinarum* strain exposed to heat shock, 247 genes were differentially expressed (55, 56). A significant fraction of these heat-responsive genes were also differentially expressed in the Δ*copR* mutant grown under copper overload conditions (22) and/or bound by CopR under optimum growth conditions (54) (63 of 247; significance by the hypergeometric distribution test, *P* ≤ 1.42 × 10^−8^; Fig. 5B and Fig. S5). Chaperones and amino acid metabolic functional categories were significantly enriched among heat-induced and CopR-repressed genes (Fig. 5B). In contrast, energy generation, translation, and transcription functions were significantly enriched among heat-repressed and CopR-induced genes (Fig. 5B). Of these 63 heat-responsive CopR-regulated genes, 6 overlapped with the list of 10 genes whose transcripts and proteins were most strongly induced in response to heat shock (56) (hypergeometric test *P* ≤ 5.0 × 10^−5^). These six genes encoded proteins required for cellular repair following heat shock (chaperones CctA and Hsp5) and protection from further damage (metalloprotein NirJ, ferritin DpsA, and anaerobic metabolic genes ArcAC). Together, these CopR-regulated gene functions are consistent with a cellular need to arrest growth to refold and regenerate degraded proteins under elevated temperatures (57).

CopR regulation of gene expression described above was tested under standard and copper overload conditions (22, 54). To validate whether CopR is also specifically required for regulation during elevated temperature, gene expression was measured by quantitative reverse transcription-PCR (qRT-PCR) in the Δ*copR* strain immediately before and 30 min after a shift from 42°C to 54°C. *cctA* expression in the Δ*copR* mutant cells was 3- to 4-fold higher under standard conditions than in Δ*ura3* parent control cells and remained elevated upon heat shock (Fig. 5C). This indicates that CopR is required for *cctA* repression under standard conditions and that relief of CopR repression is necessary for *cctA* heat induction. In contrast, 7-fold induction of *nirJ* in the Δ*ura3* strain was abrogated in the Δ*copR* mutant, indicating that CopR is an activator of this gene under heat shock (Fig. 5C). Taken together, these validation analyses suggest that CopR functions both as a global regulator of gene expression under heat shock and as a specific regulator of a copper efflux transporter during copper overload (22, 58). The connection between heat shock and copper overload has not been established in archaea; however, because both heat shock and copper overload can induce the accumulation of oxidative radicals, the transcriptional responses to these common types of cellular damage may be linked (4).

### (ii) A novel oxidative stress response function for CspD1, a conserved cold shock family protein.

The Δ*cspD1* strain exhibited the strongest growth defect of all 27 mutants under oxidative stress induced by PQ and the sixth strongest phenotype under peroxide (Fig. 2C and 3). The CspD1 sequence showed strong similarity to the cold shock family of proteins (E value of 2.30 × 10^−21^; Table S1). Cold shock domain (CSD) proteins are broadly conserved from bacteria to humans but serve diverse functions, including RNA protection, inhibition of DNA replication during oxidative stress, and transcriptional regulation under various stress conditions (59, 60). Thus, we further investigated the role of CspD1 in response to multiple stresses. We found that, although the *cspD1* gene was significantly repressed in response to high temperature (Fig. 5B) (56), the growth of the Δ*cspD1* mutant strain was indistinguishable from that of the Δ*ura3* control strain under heat and cold shock conditions (Fig. S6). In addition, *cspD1* expression was induced under copper overload conditions in a CopR-dependent manner (Fig. 5B) (22); however, the Δ*cspD1* mutant did not exhibit impaired growth under copper overload conditions (22). These data suggest either that CspD1 does not play a role in the temperature shock and copper overload responses or that CspD1 is functionally redundant under these conditions with other, as yet unknown, TFs.

10.1128/mSystems.00032-17.6 FIG S6  (A) Growth of the Δ*cspD1* mutant relative to growth of the Δ*ura3* strain under cold shock conditions. Data points represent the means for three biological replicate cultures, and error bars depict standard deviations. Only one time point is significant by *t* test. (B) The OD_Δ_ trajectory for the Δ*cspD1* mutant is not significantly different from that of the Δ*ura3* strain under heat shock. Download FIG S6, PDF file, 0.2 MB. Copyright © 2017 Darnell et al. 2017 Darnell et al. This content is distributed under the terms of the Creative Commons Attribution 4.0 International license.

**FIG 6:**
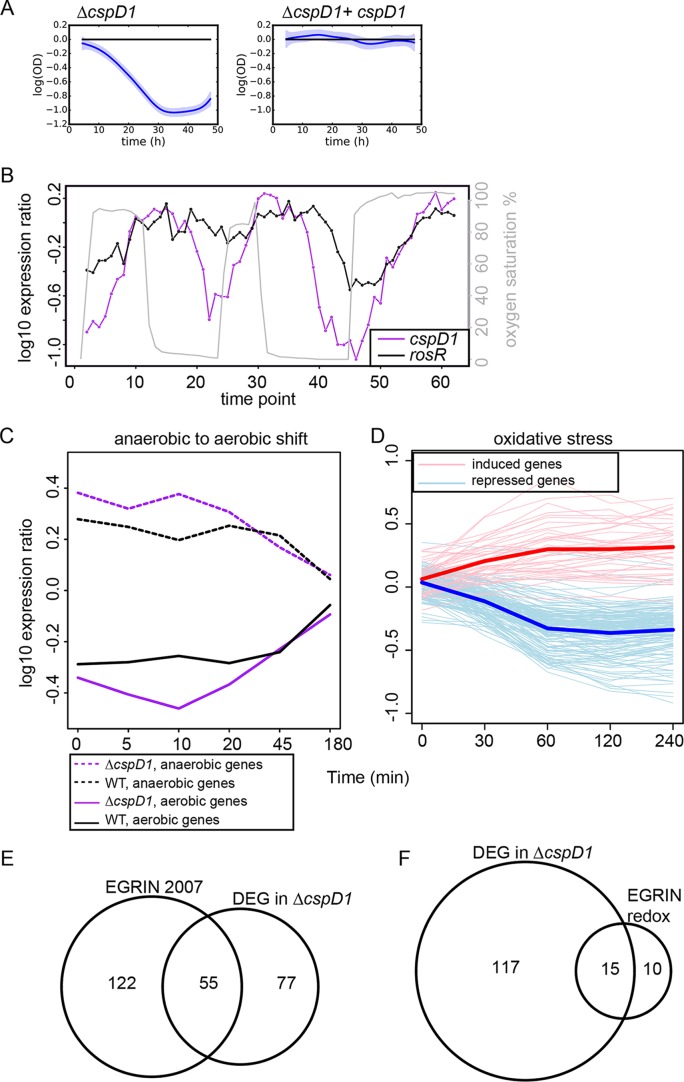
Phenotype validation: a novel oxidative stress function for the cold shock family protein CspD1. (A) Slow growth under oxidative stress conditions in the Δ*cspD1* mutant (left graph) is complemented by expression of the *cspD1* gene in *trans* (right graph). (B) Line plot depicting the expression of the *cspD1* gene (left axis) during fluctuations in oxygen concentrations (gray line, right axis). *cspD1* expression is compared to that of the gene encoding known oxidative stress regulator RosR (left axis). (C) Line plot depicting the expression of genes requiring CspD1 for appropriate dynamic expression in response to oxygen. Each line represents the mean expression value of the groups of genes indicated in the legend. Expression data and annotations for individual genes are given in Table S4. WT, wild type. (D) Expression profiles for 106 of the 132 CspD1-dependent genes differentially expressed under PQ conditions in wild-type cells (15). Thin lines represent the expression of individual genes. Thick lines show the mean of induced or repressed genes. (E) Overlap between EGRIN predictions for CspD1 target gene regulatory influences (7) and differentially expressed genes (DEG) in the Δ*cspD1* background. (F) Similar to panel E except that predictions under oxidative stress conditions are shown (15).

To further test the role of CspD1 in oxidative stress, we expressed the *cspD1* gene in *trans* on a plasmid in the Δ*cspD1* knockout background. The oxidative stress phenotype was complemented in this strain, confirming that deletion of *cspD1* was responsible for the observed phenotypes (Fig. 6A). In previously published transcriptomic data, the expression of the *cspD1* gene was strongly correlated with fluctuations in oxygen levels (1), induced 30 min after an increase in oxygen (cross-correlation $\hat{\rho }$ = 0.730; Fig. 6B). Such expression follows a pattern similar to that observed for *rosR*, which encodes a known global regulator of the oxidative stress response (16, 50). In the Δ*cspD1* strain exposed to fluctuating oxygen levels over time (see Materials and Methods), the transcription of 132 of the 660 known oxygen-responsive genes in *H. salinarum* (1) exhibited significantly altered expression (Fig. 6C and Table S4). Of these 132 genes, 89 are induced in a CspD1-dependent manner during the transition from anaerobic to aerobic conditions. According to arCOG (clusters of orthologous genes for archaea) ontology (61), these aerobic genes are significantly enriched for functions crucial for cell growth (e.g., translation; *P* ≤ 5.68 × 10^−20 ^by the hypergeometric distribution test; Table 2). In contrast, CspD1 is required to repress 43 genes under anaerobic conditions (Fig. 6C and Table 2). Of the 132 genes requiring CspD1 for appropriate expression under oxygen fluctuation, 106 are also differentially expressed under oxidative stress induced by PQ (Fig. 6D) (15).

10.1128/mSystems.00032-17.10 TABLE S4  Gene expression data for the Δ*cspD1* mutant versus wild-type *H. salinarum* under different oxygen concentrations. Download TABLE S4, XLSX file, 0.3 MB. Copyright © 2017 Darnell et al. 2017 Darnell et al. This content is distributed under the terms of the Creative Commons Attribution 4.0 International license.

**TABLE 2:** Functions significantly enriched among CspD1 target genes under fluctuating oxygen conditions

Condition	Category	*P* value^a^
Aerobic	Coenzyme transport and metabolism	2.48 × 10^−4^
	Energy production and conversion	6.33 × 10^−3^
	Translation; ribosomal structure and biogenesis	5.68 × 10^−20^

Anaerobic	Amino acid transport and metabolism	1.81 × 10^−3^

a *P* values result from the hypergeometric distribution test in arCOG categories.

The EGRIN model based on gene expression under a wide array of conditions accurately predicted a significant fraction of the 132 CspD1-dependent genes (42%; *P* ≤ 6.33 × 10^−32^ by the hypergeometric distribution test) (7) (Fig. 6E). These EGRIN-predicted genes were also significantly enriched for functions involved in ribosome biogenesis (*P* ≤ 1.8 × 10^−26^). Similarly, EGRIN predictions based solely on gene expression under oxidative stress also predicted that CspD1 regulates a significant fraction of the 132 CspD1-dependent genes (*P* ≤ 3.83 × 10^−15^) (15) (Fig. 6F). Together, these results provide validation of GRN functional predictions and phenotype analysis, implicating CspD1 in the regulation of functions critical to growth under oxidative stress and fluctuating oxygen conditions.

### Conclusions and perspectives.

Data and analyses presented here enabled the discovery of physiological roles for 17 previously uncharacterized TFs in the archaeal species *H. salinarum*. New physiological roles for previously characterized TFs were also revealed (e.g., CspD1 and CopR). This demonstrates the power of our combined high-throughput growth analysis and quantitative growth modeling for discovering unknown gene functions. The functions of a large fraction of genes and pathways remain unknown even in well-characterized model microorganisms. Recently, other high-throughput, genome-wide forward and reverse genetic approaches have made great strides in gene functional discovery, such as population genomics in *Saccharomyces cerevisiae* (62), clustered regularly interspaced short palindromic repeat interference (CRISPRi) in *Bacillus subtilis* (63), and genome-wide knockout collections in *Escherichia coli* (33, 64), as well as nontraditional model organisms such as methanogenic archaea (65). Previously we showed that Gaussian process (GP) methods for phenotype discovery are generally applicable across diverse species (35). Additionally, GPs have been shown to be useful in other domains of biological research, such as modeling genome-wide expression data (66–69). Here we extend the use of GP methods in the context of functional ANOVA to compare the growth of a large collection of strains (Fig. 2), demonstrating the promise of these methods for genome-wide functional discovery across a variety of species.

Here we directly test the predictions of computationally inferred, global GRN models such as EGRIN (7, 15). Because these and other statistical GRN inference models were inferred directly from gene expression data, the direct impact of the GRN activity on cellular physiology remained unclear. Here we show that models such as EGRIN predict not only gene expression but also the phenotypic impact of such expression (Fig. 2 and 6). In previous work, we also demonstrated the accuracy of EGRIN in predicting TF functions. For example, hypotheses generated from EGRIN predictions enabled the discovery of RosR, an archaeon-specific, novel master regulator of oxidative stress response (7, 15, 16, 50). Similarly, EGRIN model predictions regarding the cross-regulation of phosphate metabolism and methanogenesis pathways were validated by gene knockout studies in methanogens (30). Here we also observed extensive cross-regulation: each TF was important for resistance of multiple stressors, and multiple TFs played a role in surviving each stressor (Fig. 3). Such cross-regulation of gene expression has also been observed in bacteria as a means to integrate environmental cues that cooccur (e.g., heat and singlet oxygen [8, 70]) or that induce functionally related response pathways (e.g., metal homeostasis and oxidative stress [71]). Together with these previous studies, the broader investigation of 27 TF knockout phenotypes reported here demonstrates that GRN models such as EGRIN are effective tools for generating accurate hypotheses regarding TF functions. The combination of GRN modeling and phenomic validation reveals the direct impact of a complex web of regulatory interactions on cell physiology (Fig. 4 and 5). This work supports an emerging general principle that cross-regulation between TFs within the GRN enables a coordinated response to a variety of environmental stimuli.

## MATERIALS AND METHODS

### Culturing and construction of transcription factor mutants.

*Halobacterium salinarum* NRC-1 (ATCC 700922) was used as the wild-type strain background. Constructed mutants are derivatives of the Δ*ura3* strain (44), and the Δ*ura3* strain was used as the isogenic parent strain as a control in all assays. *H. salinarum* was grown routinely in complex medium (CM) (250 g/liter NaCl, 20 g/liter MgSO_4_ ⋅ 7H_2_O, 3 g/liter sodium citrate, 2 g/liter KCl, 10 g/liter peptone) supplemented with 50 μg/ml uracil to complement the uracil auxotrophy of the Δ*ura3* parent background. *E. coli* strain DH5α used for routine cloning was grown in LB containing carbenicillin (50 μg/ml) to maintain plasmids. Mutants were constructed as previously reported using the standard double-crossover counterselection method (44). Briefly, approximately 500 bp of flanking regions up- and downstream of the gene of interest were integrated into the StuI restriction site of plasmid pNBKO7 by blunt-end ligation (details of all plasmid constructs listed in Table S2 in the supplemental material). The resultant constructs were transformed into the Δ*ura3* strain and selected on CM plates containing mevinolin (10 μg/ml). The resulting merodiploid strains were then plated on CM plates containing 5-fluoroorotic acid (250 μg/ml) and uracil to remove the integrated plasmid, yielding unmarked TF deletion strains. Complementation strains were constructed using the pMTF-cmyc vector backbone (72) by isothermal Gibson assembly (73) and routinely maintained in liquid culture in CM supplemented with mevinolin (1 μg/ml). All strains were verified as described in reference 19. Genotype results are given in Fig. S1, and primer, strain, and plasmid details are given in Table S2. 

### Growth curve assays.

Cultures were pregrown in standard conditions, which were defined as growth in CM containing uracil at 42°C with shaking (225 rpm) under ambient light until early stationary phase, measured by an optical density at 600 nm (OD_600_) ≈ 2.0 (74). Each strain was then subcultured to an OD_600 _≈ 0.05 in 200 μl CM containing uracil under continuous shaking at 42°C in a Bioscreen C analysis system (Growth Curves USA, Piscataway, NJ) set to measure OD_600_ every 30 min for the duration of the 48-h experiment. Each strain was tested in at least biological quadruplicate samples, each with three technical replicates. For heat stress experiments, the temperature was shifted to 54°C at 16 h, and the elevated temperature was maintained for the remainder of the experiment. For oxidative stress experiments, hydrogen peroxide (5 mM) or paraquat (PQ) (0.333 mM) was added at the beginning of growth. For low-salinity experiments, strains were grown in CM medium containing 2.9 M NaCl. For cold growth curves (Fig. S6), cultures were pregrown in standard conditions until stationary phase, then subcultured to a starting OD_600_ of 0.1 into 5 ml of CM containing uracil and incubated at 15°C with shaking (225 rpm). Sample aliquots were taken every 24 h for 5 days to measure OD_600_.

### FANOVA growth curve model framework.

Growth data were then modeled using functional analysis of variance (FANOVA) (75), using a Bayesian approach (47). FANOVA models data as a linear combination of functional effects, where the number of effects is determined by the experiment. For example, in the case of two experimental perturbations, observations at time point *t* for effects *i* and *j* are modeled as:

(3) $$y_{i,j}(t)=\mu (t)+\alpha_{i}(t)+\beta_{j}(t)+{\left(\alpha \beta \right)}_{i,j}(t)+\varepsilon_{i,j}(t)$$

where μ_(t)_ is a mean function, α_*i*_(*t*) and β_*j*_(*t*) are the effect functions, (αβ)_*i*,*j*_ is the interaction between them, and ε_*i*,*j*_(*t*) is observation noise. Functional effects and interactions can be added and removed from the model as needed for different experimental designs. In order to make the latent effect functions identifiable, they are constrained to sum to zero:

(4) $$\overset{n_{\alpha }}{\underset{i=1}{\sum }}\alpha_{i}(t)=\overset{n_{\beta }}{\underset{j\;=\;1}{\sum }}\beta_{j}(t)=\overset{n_{\alpha }}{\underset{i\;=\;1}{\sum }}{\left(\alpha \beta \right)}_{i,j}(t)=\overset{n_{\beta }}{\underset{j\;=\;1}{\sum }}{\left(\alpha \beta \right)}_{i,j}(t)=0\;\forall t\;$$

The mean function μ(*t*) is given a GP prior directly:

(5) $$\mu (t)\sim \text{GP}(0,\kappa_{\mu }(t_{1},t_{2}))$$

In order to satisfy the identifiability constraints (equation 4), effect functions are parameterized using a set of contrast functions. For example, α_*i*_(*t*) is defined as a linear combination of the contrast functions:

(6) $$\alpha_{i}(t)=\overset{n_{\alpha }-\;1}{\underset{k\;=\;1}{\sum }}c_{ik}\times \alpha_{k}^{*}(t)=c_{i}^{T}\alpha^{*}(t)$$

where

(7) $$\alpha^{*}(t)=\{\alpha_{1}(t),\alpha_{2}(t),\ldots ,\alpha_{n_{\alpha }-\;1}(t)\}$$

Gaussian process (GP) priors were assigned to the latent functions as described in reference 47. GPs place a distribution on a continuous function, any finite number of observations of which are distributed as multivariate normal (76). Each GP prior is parameterized by a mean function *m*(*x*) and a covariance function, κ(*x*_1_,*x*_2_). All prior mean functions in this analysis were set identically to zero, as is standard. Covariance functions were modeled using radial basis functions (RBFs):

(8) $$\kappa (x_{1},x_{2})=\sigma^{2}\times \exp\left(\frac{{-||x_{1}-x_{2}||}^{2}}{\ell }\right)$$

where σ^2^ and *ℓ* are hyperparameters defining the variance and length scale of the GP, respectively. The variance σ^2^ determines the magnitude of variability for a given GP prior distribution, with higher variances leading to more-variable functions. The length scale parameter controls the rate of decay of covariance between two time points, and larger length scales place higher probability on smoother (slower changing) functions.

All contrast functions for a given effect are defined by a shared GP prior:

(9) $$\alpha_{i}^{*}(t)\sim GP(0,\kappa_{\alpha })$$

where 1 ≤ *i* ≤ *n*_α_ − 1. Kernel hyperparameters were given noninformative priors. Posterior quantities were obtained by Markov chain Monte Carlo (MCMC) simulation. Sampling of the contrast functions was accomplished using Gibbs sample updates (47). Kernel hyperparameters were sampled via slice sampling (77).

### Determination of significant growth phenotypes.

All raw growth data (Table S3) were first normalized to the log_2_ scale. The first 4 h of growth (less than one generation) were removed because technical and instrument variability is often observed during this time frame. Growth data were then grouped by strain and experimental design (standard growth, 0.333 mM PQ, 2.9 M NaCl, or heat shock), referred to as the condition. Growth data corresponding to the same condition were scaled by a fixed value so the mean of the condition at the earliest time point was equal to zero. The growth data for *H. salinarum* TF mutants under various stress conditions was modeled with GP FANOVA as described below.

### (i) Standard, oxidative stress, and osmotic stress conditions.

Growth data were modeled with two effects corresponding to strain and experimental design. The strain effect, α_*i*_, varied from *i* = 1 to *i* = 28, where *i* = 1 corresponded to the Δ*ura3* parent strain and *i* > 1 corresponded to one of the 27 mutant strains. The experimental design effect, β_*j*_ (1 ≤ *j* ≤ 4), represented one of four experimental designs: standard growth (*j* = 1), low-osmolarity stress (*j* = 2), PQ (*j* = 3), or peroxide stress (*j* = 4). Interactions between the two effects were also modeled to determine the strain-specific responses to each of the stress effects. The full GP FANOVA model for the analysis was then the same as in equation 3 and was estimated using the GP FANOVA model as described above. Variation between phenotypes arising from separate batches of experiments were controlled by adding a specific batch function, γ(*t*). 

### (ii) Heat shock.

Growth data for heat shock conditions was modeled using a GP FANOVA modeling the individual effect of strain, α_*i*_. This model has the form

(10) $$y_{i}(t)=\mu (t)+\alpha_{i}(t)+\varepsilon_{i}(t)$$

All metrics for the heat shock condition (OD_Δ_ and$||{\text{OD}}_{\Delta }||$, described below) were computed starting at the 16-h time point, the beginning of the shock. Additionally, the difference between the Δ*ura3* parent and mutant strain was subtracted from all metrics, which removes any confounding differential growth that occurred between the two strains prior to the shock initiation.

### (iii) Δ*copR* complementation.

Complementation was modeled as the combined effect of strain (α_*i*_, Δ*ura3* or Δ*copR*), empty vector (β), and presence or absence of the *copR* complementation on the plasmid (γ). The FANOVA model for this condition is then

(11) $$y_{i,j,k}(t)=\mu (t)+\alpha_{i}(t)+\beta_{j}(t)+\gamma_{k}(t)+\varepsilon_{i,j,k}(t)$$

There are two states for each effect, and we are interested in estimating the fixed effect of the *copR* complementation only under the heat stress condition starting at 16 h, so no interactions between condition and strain were needed in this model.

### (iv) Δ*cspD1* complementation.

Complementation of Δ*cspD1* in H_2_O_2_ was modeled with an extension of the model for Δ*copR* in heat shock (equation 11). Functions for strain (α), condition (β), and their interaction (α,β) are included, as in equation 3. In addition, a function is included to model the presence or absence of the empty vector γ, as well as the presence or absence of Δ*cspD1* on the plasmid (δ). In this case, we are specifically interested in the complementation provided by the plasmid-expressed *cspD1* to the Δ*cspD1* strain in the H_2_O_2_ condition, so we modeled the interaction of this strain with the condition (β,δ):

(12) $$y_{i,j,k,l}=\mu +\alpha_{i}+\beta_{j}+\gamma_{k}+\delta_{l}+{\left(\alpha \beta \right)}_{i,j}+{\left(\beta \delta \right)}_{j,l}$$

### (v) Significance test.

Two metrics were used to assess the significance of the difference between the growth of each mutant versus the parent strain under each condition.

The first metric, OD_Δ_, was first computed for each strain under standard conditions as

(13) $${\text{OD}}_{\Delta }(t)=y_{i,1}(t)-y_{1,1}(t)$$

(14) $${\text{OD}}_{\Delta }(t)=\alpha_{i}(t)-\alpha_{1}(t)+{\left(\alpha \beta \right)}_{i,1}(t)-{\left(\alpha \beta \right)}_{1,1}(t)$$

where *i* represents the strain of interest. This represents the difference between the parent strain and TF mutant strain under standard conditions. When comparing mutant and parent strain under nonstandard conditions, the function described in equation 14 is used as the control of the difference between the parent and mutant strains. This leads to the formulation of OD_Δ_ under stress conditions as

(15) $${\text{OD}}_{\Delta }(t)=[y_{i,j}(t)-y_{1,j}(t)]-[y_{i,1}(t)-y_{1,1}(t)]$$

(16) $${\text{OD}}_{\Delta }(t)={\left(\alpha \beta \right)}_{i,j}(t)-{\left(\alpha \beta \right)}_{1,j}(t)-{\left(\alpha \beta \right)}_{i,1}(t)+{\left(\alpha \beta \right)}_{1,1}(t)$$

which is the difference between strain *i* and the parent strain under condition *j*, normalized by the difference between the two strains under standard conditions. In this formulation, if the difference between the parent strain and mutant strain is the same under both standard conditions and one of the stress conditions, OD_Δ_ will be close to zero under the stress condition for that strain. Both formulations of OD_Δ_ in equation 14 and equation 16 use effect functions estimated by the GP FANOVA model and can therefore be calculated from the model posterior samples. The posterior distribution of OD_Δ_ for each strain under a given condition was used to determine where a mutant strain and the Δ*ura3* parent were significantly different as a function of time. Ninety-five percent credible intervals for OD_Δ_ were constructed using the posterior samples of the GP FANOVA model, and any time point where zero was not included in the interval was considered significantly different from the parent strain.

To obtain an overall test to rank order the significance of the phenotype under a specific condition, a second metric was derived from OD_Δ_ representing the overall magnitude of growth difference between the parent and mutant strains. This metric, $||{\text{OD}}_{\Delta }||$, was calculated as in equation 2 which represents the magnitude of OD_Δ_ over the entire growth curve. Larger values of $||{\text{OD}}_{\Delta }||$ indicate growth phenotypes that deviate most from that of the parent strain. This metric can be used to directly compare different mutant strains for the overall significance of their growth phenotype.

Phenotype networks (Fig. 3) were constructed and visualized using Cytoscape (78). The $||{\text{OD}}_{\Delta }||$ cutoff of 0.337 was used to enable comparison across mutants and across conditions. This cutoff was chosen to (i) exclude mutants whose maximum or minimum OD_Δ_ value was inside the 95% confidence interval and (ii) exclude mutants outside the top 10% of $||{\text{OD}}_{\Delta }||$ values under standard conditions (Fig. 2).

### Transcript quantitation with qRT-PCR.

Samples were harvested at mid-log phase (OD_600_ ≈ 0.4) and 30 min after the shift to 54°C. RNA was purified using an Absolutely RNA Miniprep kit (Agilent Technologies, Santa Clara, CA), and 500 ng was tested for DNA contamination by PCR with *Taq* DNA polymerase (Thermo Scientific, Grand Island, NY) and for integrity with a 2100 bioanalyzer (Agilent Technologies, Santa Clara, CA). Quantitative reverse transcription-PCR (qRT-PCR) primers (Integrated DNA Technologies, Coralville, IA) are listed in Table S2. The iTaq universal SYBR green one-step kit (Bio-Rad, Hercules, CA) was used in 20-μl reaction mixtures according to the manufacturer’s instructions. Quantitative analysis was performed in a Bio-Rad real-time thermal cycler (Bio-Rad, Hercules, CA). Expression relative to the *VNG1756G* reference locus (18) was calculated using the ΔΔ*C*_*T*_ method (18, 79).

### CspD1 validation experiments. (i) Transcriptomic analysis of the Δ*cspD1* mutant exposed to different oxygen levels.

Although these data were published previously as part of a larger gene regulatory network study (43), here we report the details of the experiment and specific analysis of the effect of the Δ*cspD1* deletion on gene expression. Briefly, the Δ*cspD1* mutant and Δ*ura3* strain were grown to mid-logarithmic phase in batch mode in a New Brunswick BioFlo100 modular benchtop fermentor (New Brunswick Scientific) in CM medium as described in reference 1. At mid-log phase, oxygen sparging and agitation were stopped to induce anoxia. The cultures were incubated anaerobically overnight, then oxygen was sparged, and RNA was collected at time points immediately prior to the addition of oxygen and 5, 10, 20, 45, and 180 min afterwards. RNA extraction, microarray hybridization, scanning, and preprocessing were conducted as described in reference 1. Data were analyzed using a modified ANOVA in the Statistical Analysis of Microarrays (SAM) package in the TM4 freeware program (80) to determine the final list of genes differentially expressed in response to oxygen and *cspD1* deletion (Table S4).

### (ii) Analysis of PQ transcriptomic data.

Wild-type *H. salinarum* NRC-1 cultures in mid-logarithmic phase were exposed to 4 mM PQ, and transcriptomics by microarrays were monitored immediately prior to PQ exposure and 30, 60, 120, and 240 min afterward. These data were previously described in reference 15. In this study, we performed hierarchical clustering of these data in the TM4 program to determine which genes were induced and which genes were repressed in response to PQ. These PQ-responsive gene sets were then filtered to include only the 132 genes differentially expressed in the Δ*cspD1* mutant (Fig. 6C) and plotted in the R environment base package (Fig. 6D) (81).

### (iii) Comparison of CspD1 target genes from EGRIN predictions with transcriptomic validation data.

EGRIN predictions of CspD1-regulated genes were filtered in Cytoscape (78). For predictions from reference 7, cluster residuals of <0.4 and CspD1 target gene edge weights of ≥0.2 were considered significant; for predictions from reference 15, residuals of <0.4 and edge weight of >0.1 were considered significant. These criteria are consistent with those used in the original EGRIN publications. The significance of the overlap between genes in resultant clusters and genes differentially expressed in the Δ*cspD1* mutant (Table S4) was determined using the hypergeometric distribution.

### General statistical methods used for analysis of validation experiments.

OD_Δ_ phenotype correlations and their significance described in the legend to Fig. 4 were calculated in R using the rcorr() function in the Hmisc package (82) and visualized using the corrplot package (83). Significance of overlap between disparate gene lists was calculated by the hypergeometric distribution test. For all gene lists, enrichment in arCOG functional categories (61) relative to the genomic background was calculated by the hypergeometric distribution test as described previously (84, 85).

### Data availability.

Raw and normalized microarray data and metadata from the Δ*cspD1* mutant exposed to oxygen are freely accessible at the NCBI Gene Expression Omnibus (GEO) database (86) (https://www.ncbi.nlm.nih.gov/geo/) under accession numbers GSE97933 and GPL22925. Data for PQ gene expression are accessible in the GEO database via accession GSE17515. Phenotyping data generated in this study are available in the supplemental material (Table S3). The code repository for the FANOVA model is freely available at https://github.com/ptonner/hsalinarum_tf_phenotype. Code for determining enrichment in arCOG functional groups is freely available at https://github.com/amyschmid/histone_arCOG (85).
