# Supplementary material for: Systematic Discovery of Archaeal Transcription Factor Functions in Regulatory Networks through Quantitative Phenotyping Analysis
Source: mSystems. 2017 Sep 19;2(5):e00032-17. doi: 10.1128/mSystems.00032-17 (PMC5605881; doi:10.1128/mSystems.00032-17)
Supplement: FIG S1 [file sys004172130sf1.pdf]

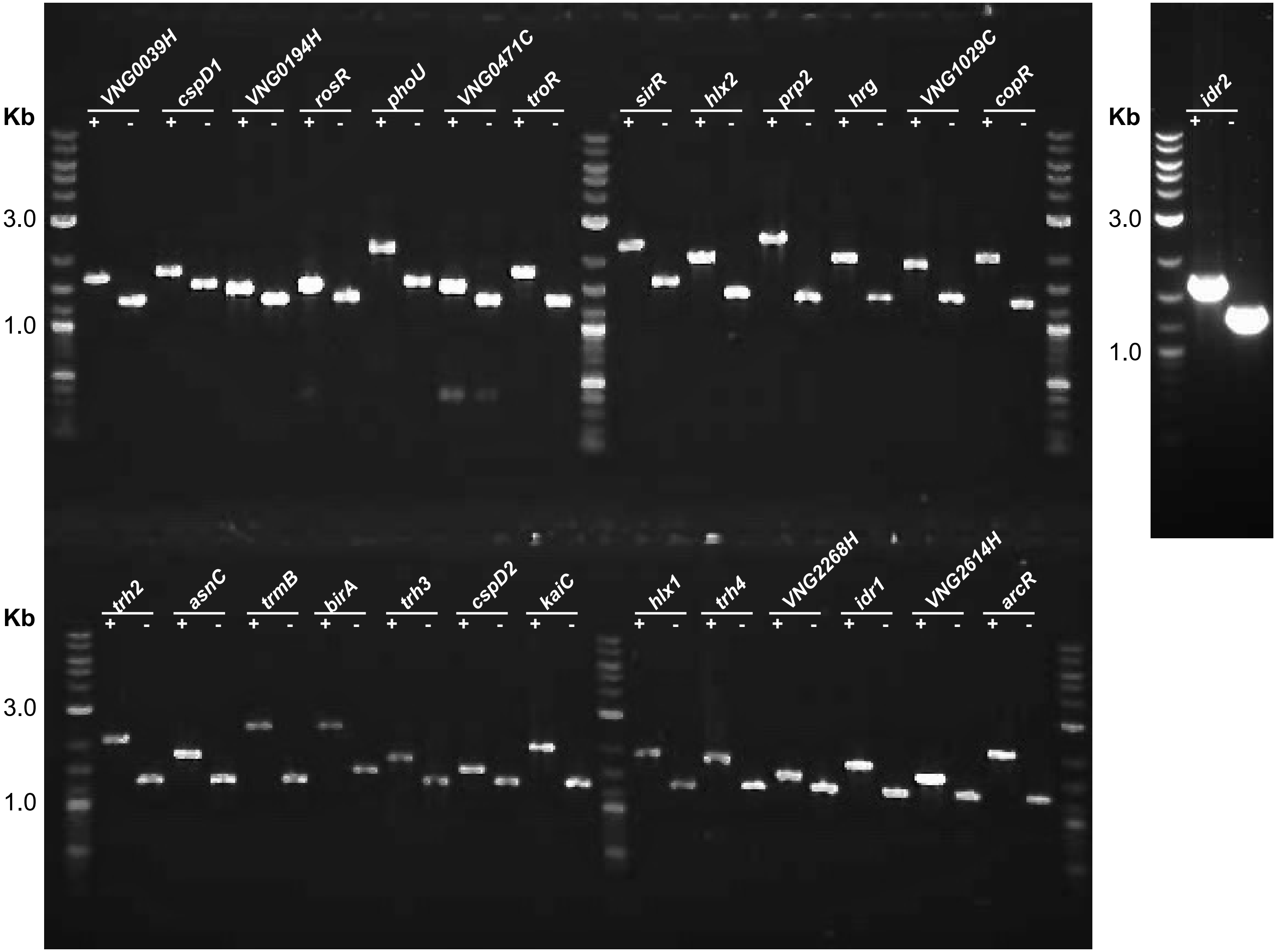

Supplementary Figure S1. PCR confirmation of TF mutants. See Supplementary Table S2 for the primers and PCR conditions. All PCR products are diluted to correct for DNA concentration and the resulting band brightness. +, PCR product from Δ*ura3* strain. -, product from mutant strain indicated at top. Marker sizes indicated at left.
