# Supplementary figures and images for: Systematic Discovery of Archaeal Transcription Factor Functions in Regulatory Networks through Quantitative Phenotyping Analysis
Source: mSystems. 2017 Sep 19;2(5):e00032-17. doi: 10.1128/mSystems.00032-17 (PMC5605881; doi:10.1128/mSystems.00032-17)

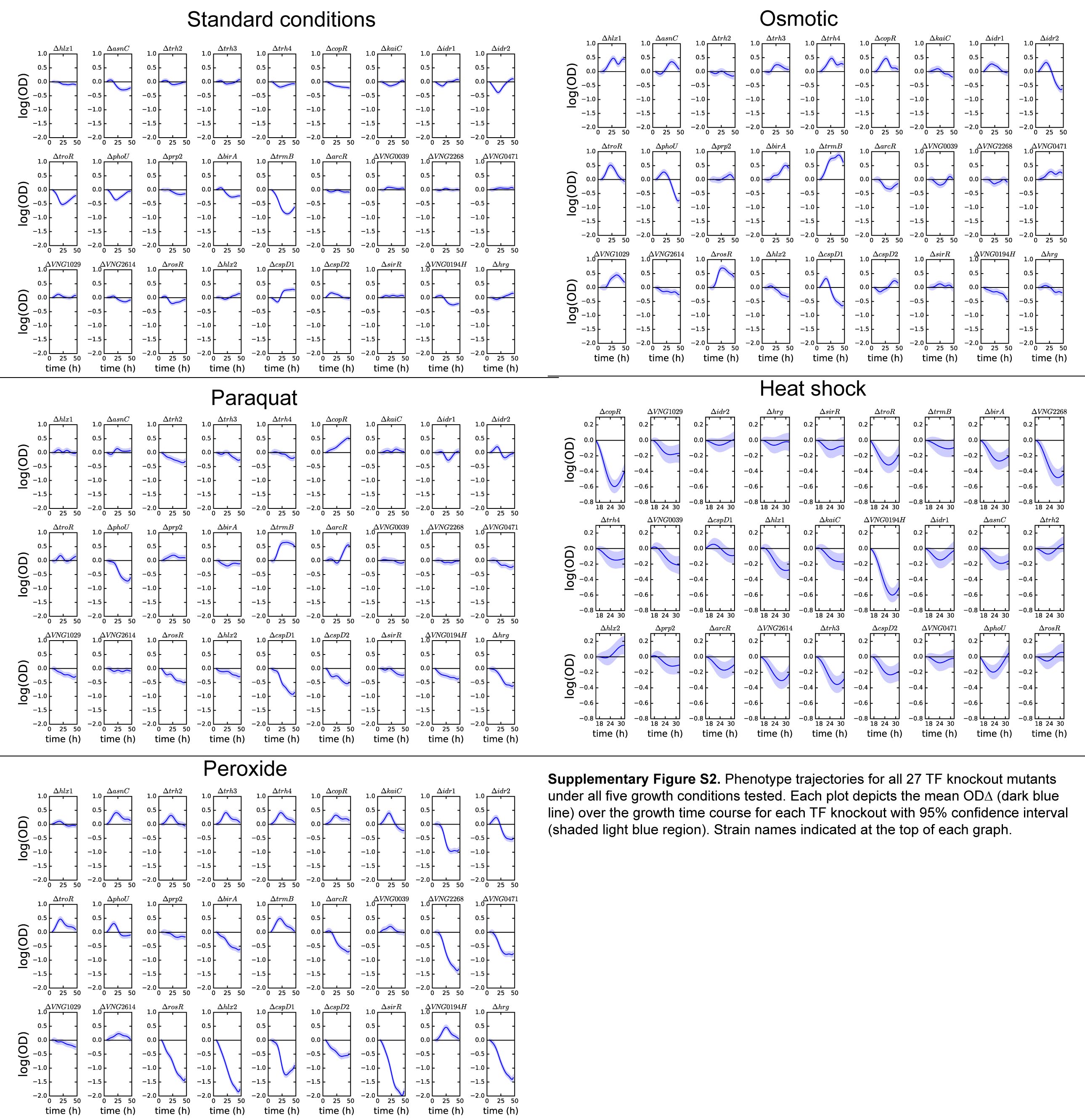

Supplement: FIG S2 [file sys004172130sf2.jpg]
