# Supplementary material for: Systematic Discovery of Archaeal Transcription Factor Functions in Regulatory Networks through Quantitative Phenotyping Analysis
Source: mSystems. 2017 Sep 19;2(5):e00032-17. doi: 10.1128/mSystems.00032-17 (PMC5605881; doi:10.1128/mSystems.00032-17)
Supplement: FIG S3 [file sys004172130sf3.pdf]

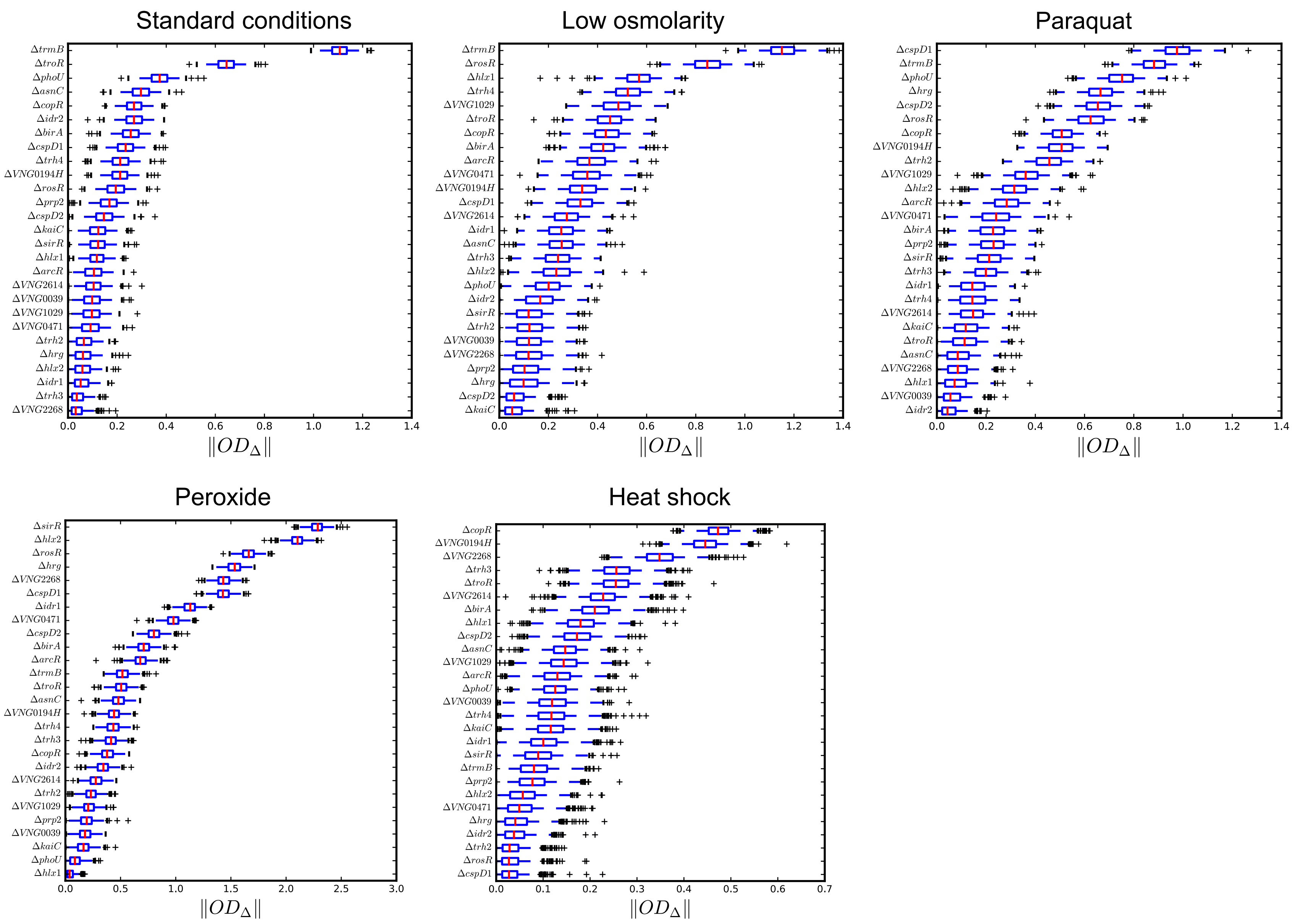

Supplementary Figure S3. Distribution of ||ODΔ|| values for all mutants across conditions. Upper and lower boundaries of blue boxes in each graph represent 1st and 3rd quartiles, respectively. Red lines represent median values, which were used to determine edge widths in phenotype network in main text Figure 3.
