# Supplementary material for: Systematic Discovery of Archaeal Transcription Factor Functions in Regulatory Networks through Quantitative Phenotyping Analysis
Source: mSystems. 2017 Sep 19;2(5):e00032-17. doi: 10.1128/mSystems.00032-17 (PMC5605881; doi:10.1128/mSystems.00032-17)
Supplement: FIG S4 [file sys004172130sf4.pdf]

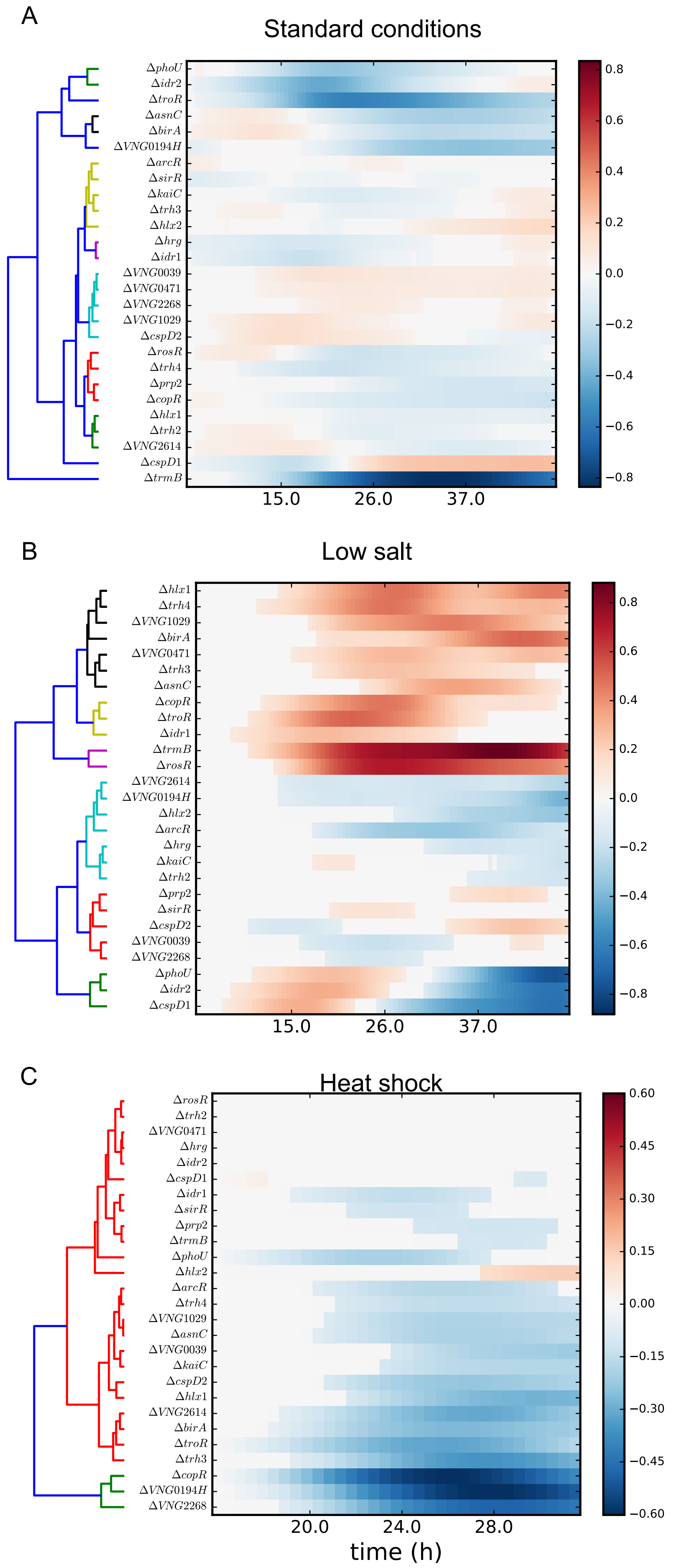

Supplementary Figure S4. Heat maps of clustering of ODΔ growth trajectories of all 27 mutants under standard (A), low salt (B), and heat shock (C) conditions. Color scale of ODΔ values shown at right for each condition. Dendrograms result from heirarchical clustering growth trajectories. Colors indicate coherent clusters at the tree height cutoff indicated where blue branches end.
