# Supplementary material for: Systematic Discovery of Archaeal Transcription Factor Functions in Regulatory Networks through Quantitative Phenotyping Analysis
Source: mSystems. 2017 Sep 19;2(5):e00032-17. doi: 10.1128/mSystems.00032-17 (PMC5605881; doi:10.1128/mSystems.00032-17)
Supplement: FIG S6 [file sys004172130sf6.pdf]

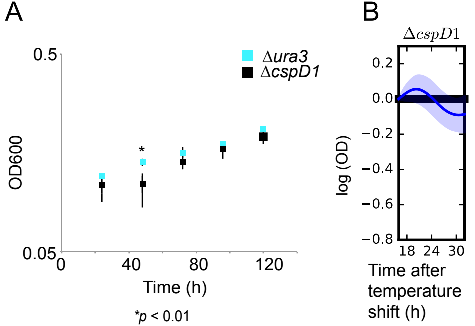

Supplementary Figure S6. (A) Growth of Δ*cspD1* relative to that of Δ*ura3* under cold shock conditions. Data points represent the mean of three biological replicate cultures and error bars depict standard devation. Only one time point is significant by t-test.

(B) Δ*cspD1* ODΔ trajectory is not significantly different than that of Δ*ura3* under heat shock.
