# Supplementary material for: Systematic Discovery of Archaeal Transcription Factor Functions in Regulatory Networks through Quantitative Phenotyping Analysis
Source: mSystems. 2017 Sep 19;2(5):e00032-17. doi: 10.1128/mSystems.00032-17 (PMC5605881; doi:10.1128/mSystems.00032-17)
Supplement: FIG S5 [file sys004172130sf5.pdf]

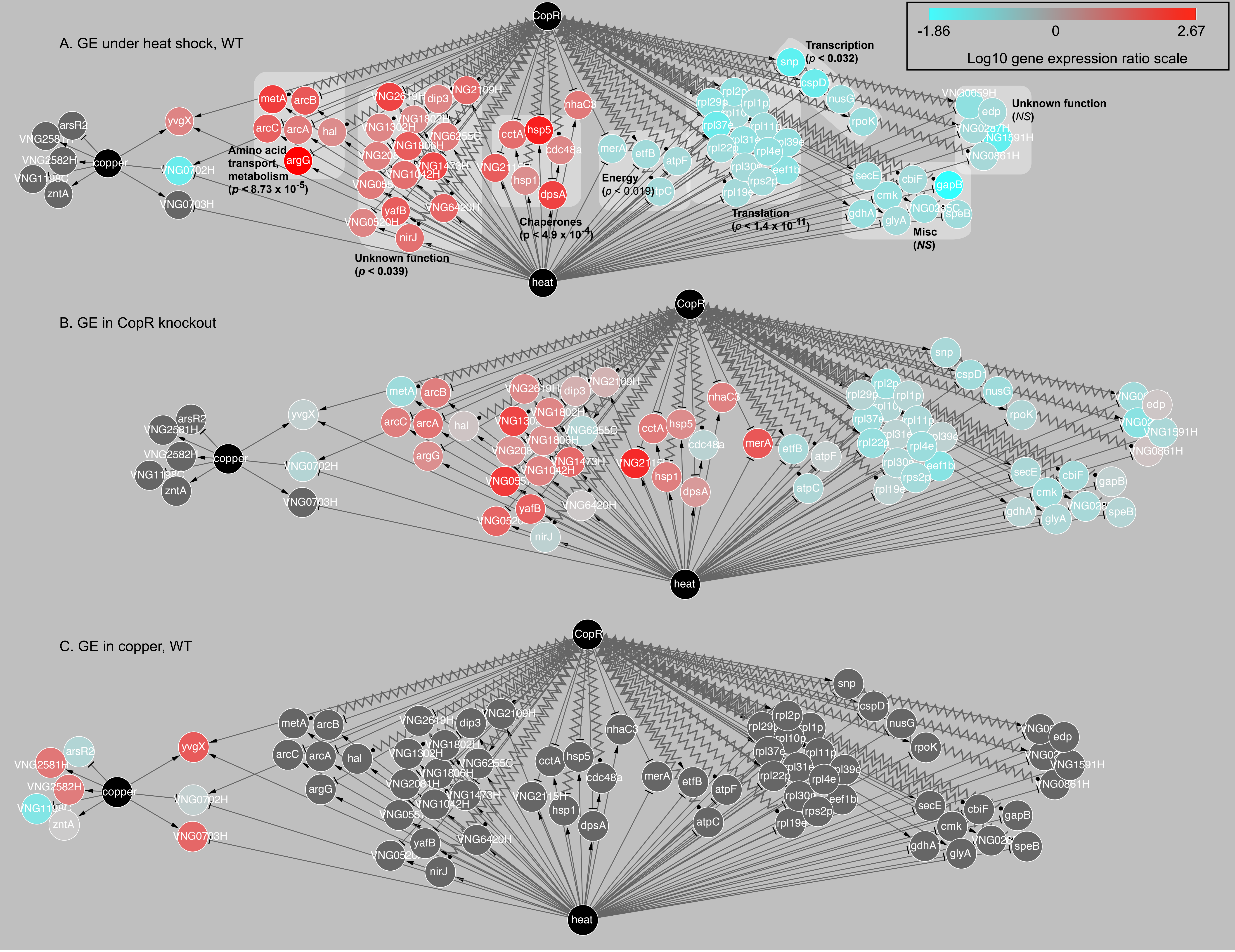

Supplementary Figure S5. Cytoscape network graphs depicting the expression of genes whose expression is affected by heat shock, copper, and deletion of *copR*. (A) Colors represent expression of genes in wild type cells under heat shock. Levels of expression are as in the color legend. Grey nodes are not differentially expressed. Edges are as in main text Figure 4. Grey boxes surrounding groups of nodes represent arCOG functional groups with enrichment significance *p*-values provided. Categories and *p*-values are consistent across subpanels. (B) Colors of nodes represent expression of genes in Δ*copR* strain. (C) Colors of nodes represent gene expression under copper overload conditions in wild type cells.
